# Fully automated detection and segmentation of meningiomas using deep learning on routine multiparametric MRI

**DOI:** 10.1007/s00330-018-5595-8

**Published:** 2018-06-25

**Authors:** Kai Roman Laukamp, Frank Thiele, Georgy Shakirin, David Zopfs, Andrea Faymonville, Marco Timmer, David Maintz, Michael Perkuhn, Jan Borggrefe

**Affiliations:** 1 Institute for Diagnostic and Interventional Radiology, University Hospital Cologne, Cologne, Germany; 2 Philips Research, Aachen, Germany; 3 Department of Neurosurgery, University Hospital Cologne, Cologne, Germany

**Keywords:** Meningioma, Brain neoplasms, Magnetic resonance imaging, Machine learning, Artificial intelligence

## Abstract

**Objectives:**

Magnetic resonance imaging (MRI) is the method of choice for imaging meningiomas. Volumetric assessment of meningiomas is highly relevant for therapy planning and monitoring. We used a multiparametric deep-learning model (DLM) on routine MRI data including images from diverse referring institutions to investigate DLM performance in automated detection and segmentation of meningiomas in comparison to manual segmentations.

**Methods:**

We included 56 of 136 consecutive preoperative MRI datasets [T1/T2-weighted, T1-weighted contrast-enhanced (T1CE), FLAIR] of meningiomas that were treated surgically at the University Hospital Cologne and graded histologically as tumour grade I (*n* = 38) or grade II (*n* = 18). The DLM was trained on an independent dataset of 249 glioma cases and segmented different tumour classes as defined in the brain tumour image segmentation benchmark (BRATS benchmark). The DLM was based on the DeepMedic architecture. Results were compared to manual segmentations by two radiologists in a consensus reading in FLAIR and T1CE.

**Results:**

The DLM detected meningiomas in 55 of 56 cases. Further, automated segmentations correlated strongly with manual segmentations: average Dice coefficients were 0.81 ± 0.10 (range, 0.46-0.93) for the total tumour volume (union of tumour volume in FLAIR and T1CE) and 0.78 ± 0.19 (range, 0.27-0.95) for contrast-enhancing tumour volume in T1CE.

**Conclusions:**

The DLM yielded accurate automated detection and segmentation of meningioma tissue despite diverse scanner data and thereby may improve and facilitate therapy planning as well as monitoring of this highly frequent tumour entity.

**Key Points:**

*• Deep learning allows for accurate meningioma detection and segmentation*

*• Deep learning helps clinicians to assess patients with meningiomas*

*• Meningioma monitoring and treatment planning can be improved*

**Electronic supplementary material:**

The online version of this article (10.1007/s00330-018-5595-8) contains supplementary material, which is available to authorized users.

## Introduction

Meningiomas are neoplasms originating from meningothelial cells and are among the most common intracranial neoplasms with an incidence of 0.9% in routine brain magnetic resonance imaging (MRI) [1–5]. Almost one-third of primary intracranial lesions are meningiomas [2]. According to the World Health Organization (WHO), the lesions are graded as benign (grade I), atypical (grade II) or anaplastic (grade III) [1, 6, 7]. The histological grading allows for the prediction of biological behaviour and prognosis of meningiomas. There have been detailed studies showing that grade II and III meningiomas are associated with increased risk of recurrence, invasiveness and aggressiveness [3, 8, 9].

MRI is the key method for diagnosis and characterisation of meningiomas, resection planning, therapy decisions and monitoring of therapy [5, 7, 10]. Typical meningiomas occur sessile or lentiform in shape and are sharply circumscribed showing wide dural attachments. They have a strong laminar contrast enhancement and are usually isointense to hyperintense in T2-weighted and fluid-attenuated inversion recovery (FLAIR) images. Apparent diffusion coefficient (ADC) values may differ significantly among meningiomas and are often isointense to normal brain tissue [7]. Peritumoural oedema of the brain parenchyma may be present, especially when meningiomas show greater tumour volumes [7]. Atypical and anaplastic meningiomas present with larger tumour volumes compared to benign meningiomas [1, 11]. Further, higher meningioma grades show faster tumour growth [1]. However, there is no clear radiological criteria so far that can reliably distinguish grade I and II meningiomas. Anaplastic grade III meningiomas present differently on MRI and are often irregularly shaped [5].

To the best of our knowledge, there are no studies regarding the fully automated detection and segmentation of meningiomas to date. As tumour progression of meningiomas is commonly slow, multifocal and occurring in different directions, an automated detection of meningiomas might facilitate and improve image reading. Regarding the manual volumetric assessment of meningiomas in MRI, it has been shown that three-dimensional assessments provide an increased sensitivity for the detection of tumour progression in comparison to two-dimensional assessments [1, 12]. Therefore, the volumetric evaluation of meningiomas is superior to traditional diameter methods when assessing tumour growth but is time-consuming [1, 12, 13]. Further, volumetric assessment of MR images of the brain is often conducted in routine image assessments and necessary for many neurological diseases, such as brain tumours [14].

In contrast, automated detection and segmentation of meningiomas in MRI may be performed as pre-processing before reading the images, possibly allowing for a more detailed analysis of tumour volumes and further multiparametric image analysis. Furthermore, automated tumour segmentation and evaluation may lead to an increased robustness and reliability due to reduced inter-reader bias [14]. As the tumour volume at primary diagnosis correlates with recurrence rates [1, 12], a precise volumetric assessment could help to differentiate between meningioma grades. However, the correlation between growth and histological grading is vague and has to be further evaluated [1, 11, 12].

Automatic brain tumour segmentation algorithms should address several challenges to be reliable, such as anatomical variations, varying imaging data due to different MRI scanners as well as variations in scanner parameters. Further, pathologies such as brain tumours vary strongly in their presentation [5, 14]. The technological advancements of deep-learning models (DLMs) led to significant improvements regarding the automated tumour detection and the technology is currently on the verge of being used in clinical routine [13, 15, 16]. DLMs work with multiple processing layers and levels of abstractions resulting in deep convolutional neural networks that need a larger amount of training data for extraction of a complex hierarchy of features by its self-learning abilities [14, 17, 18]. A neural network architecture consists of different layers for convolution, pooling, and classification [14]. The necessary training data and segmentation “gold standard” is usually obtained by manual segmentations. For manual brain tumour segmentation high intra- and inter-rater variability between 20-30% has been reported [14, 19]. Besides deep learning other (semi-)automated methods have been used for brain tumour segmentation, especially for most common intracranial neoplasms, i.e. meningiomas and gliomas. For semi-automated segmentations, various approaches have been applied, such as region growing, random walker, non-negative matrix factorisation, fuzzy clustering and livewire algorithm [20–24]. Automated tumour volume definition has also been applied in post-radiation patients using an algorithm that is based on the Chan-Vese active contour method and patient-specific intensities [25].

The purpose of this study was to investigate the reliability of automated detection and segmentation of grade I and II meningiomas using a deep learning model on routine multiparametric MRI data from diverse scanners including referring institutions.

## Materials and methods

### Patients

**Fig. 1:**
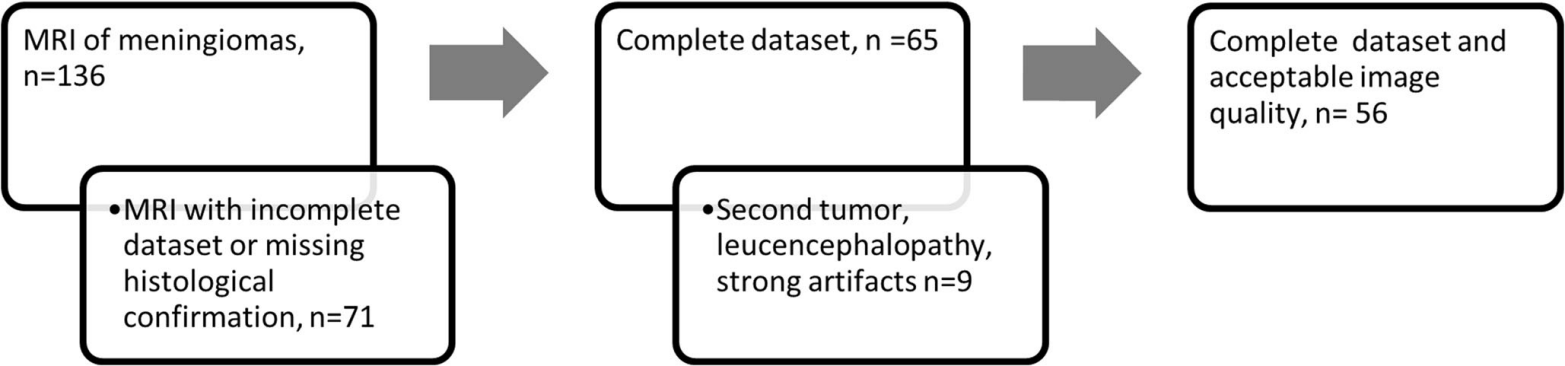
Patient selection. Patients were excluded due to an incomplete dataset, prevalent second tumour, leukoencephalopathy or severe artefacts. Fifty-six patients fulfilled all requirements and could be integrated in further machine-learning analyses

This study was approved by the local institutional review board. One hundred and thirty-six patients that were referred to the University Hospital of Cologne from January 2012 to May 2016 for treatment of meningiomas were included in this retrospective study (Fig. 1). All diagnoses were confirmed histologically according to the guidelines of the WHO [6, 8]. Only patients with a complete available MRI dataset before treatment were included, with T1- and T2-weighted, FLAIR and T1-weighted contrast-enhanced (T1CE) MRI sequences being defined as necessity for study inclusion. Slice thickness varied from 1.2 to 6 mm. Nine patients were excluded due to (1) a prevalent second tumour entity (*n* = 3), (2) severe leukoencephalopathy impairing the FLAIR tumour segmentation (Fazekas III, *n* = 4) and (3) strong artefacts due to patient movement (*n* = 2). Fifty-six patients fulfilled all criteria and were included in this study.

### MRI

All scans were conducted for clinical indications. The MRI acquisitions were performed on diverse scanners from referring institutions (*n* = 37; 1.0, 1.5, 3.0 T; (1) Siemens MRI models: Avanto, Espree, Aera, Verio, Essenza (Siemens, Erlangen, Germany), (2) Toshiba Titan (Toshiba, Tokio, Japan) and (3) Philips MRI models: Panorama, Intera, Achieva, Ingenia (Philips Healthcare, Best, The Netherlands) and on local scanners from the University Hospital of Cologne [*n* = 19, Philips models: Achieva, Ingenia, Intera; 1.5 and 3.0 T (Philips Healthcare, Best, The Netherlands)]. MRI scan parameters are given in Supplementary Table S1. At the University Hospital of Cologne, for T1CE MR images patients were injected intravenously with gadolinium (Dotarem; Guerbet, Roissy, France: 0.5 mmol/ml, i.e. 1 ml = 279.32 mg gadoteric acid = 78.6 mg gadolinium) with a concentration of 0.1 mmol/kg body weight. The contrast medium applications in the referring institutions were not standardised.

### Manual segmentation

Manual segmentation (semi-automated) was performed by two radiologists in a consensus reading using IntelliSpace Discovery (Philips Healthcare, Best, The Netherlands). Contrast-enhancing tumour and FLAIR isointense to hyperintense tumour as well as surrounding hyperintense oedema were defined as tumour volume and segmented separately in T1CE and FLAIR images. Total tumour volume (TTV) was defined as the union of tumour volumes in T1CE and FLAIR, including solid contrast-enhancing tumour parts, surrounding oedema in FLAIR and if present tumour necrosis. T1CE tumour volume was defined as contrast-enhancing tumour volume in T1CE images.

### Automated deep learning-based segmentation

The DLM for automated detection and segmentation was trained on an independent dataset of 249 glioma cases. The DLM preforms voxel-wise classifications of four tumour classes (oedema, contrast-enhancing tumour, necrosis, non-enhancing tumour) as defined in the BRATS benchmark [26].

MR images were pre-processed with established tools (SPM8, Wellcome Trust Centre for Neuroimaging, London, UK; Intellispace Discovery, Philips Healthcare, Best, The Netherlands) before feeding into automatic segmentation. The processing pipeline included (1) bias field correction, (2) co-registration, (3) skull stripping, (4) resampling to isotropic resolution of 1 × 1 × 1 mm^3^ and (4) normalisation to zero-mean and standard deviation of one. The DLM was based on the DeepMedic architecture [15] using a deep 3D convolutional neural network, followed by a 3D fully connected network to remove false positives. The 3D convolutional neural network included two pathways that apply different image resolution to capture both short and long-range characteristics of the tumour appearances. Extracted tumour volumes for analysis were TTV as well as T1CE TV.

### Statistical analysis

Statistical analyses were conducted using JMP Software (V12; SAS Institute, Cary, USA). Quantitative results are displayed as mean (± standard deviation). Wilcoxon signed rank test was used for the determination of statistical differences. Statistical significance was set to *p* < 0.05.

To evaluate automatic segmentation, the resulting tumour volumes were compared to the manual ground truth annotations. For TTV and T1CE TV, segmentations were compared with respect to volume and voxel-wise accuracy. As before, the accuracy was computed as overlap of ground truth segmentation S1 and model segmentation S2 using the Dice coefficient (similarity index) [27]:

$$ \mathrm{DSC}\left({S}_1,{S}_2\right)=\frac{2\mid {S}_1\cap {S}_2\mid }{\left|{S}_1\right|+\left|{S}_2\right|} $$

## Results

### Patients

The 56 adult patients included 28 women and 28 men with a mean age of 59.1 ± 13.7 years (range, 33-86 years). Thirty-eight patients had a grade I meningioma and 18 patients a grade II meningioma, including meningiomas of the falx (*n* = 6), convexity (*n* = 24), sphenoid wing (*n* = 9), olfactory groove (*n* = 4), suprasellar (*n* = 2), posterior fossa (*n* = 10) and with attachment to the sinus (*n* = 1).

Mean TV from manual segmentations in T1CE was 30.9 ± 25.9 cm^3^ and TTV as the union of tumour volume in T1CE and FLAIR from manual segmentation was 74.0 ± 67.2 cm^3^. TV by automated detection was smaller: TV in T1CE was 22.8 ± 18.8 cm^3^ and TTV was 67.9 ± 58.8 cm^3^. Additional detailed TV data sorted after localisation are given in Table 1.

**Table 1:** Tumour volumes listed after different localisations

Localisation	Number	Manual segmentations		Automated segmentations	
		TTV	TV in T1CE	TTV	TV in T1CE
Falx	6	104.8 ± 58.9	39.6 ± 12.7	90.0 ± 50.2	32.6 ± 16.3
Convexity	24	56.2 ± 53.5	29.0 ± 28.2	53.8 ± 49.5	21.3 ± 18.7
Sphenoid wing	9	91.8 ± 91.1	31.9 ± 28.6	84.8 ± 83.4	18.3 ± 18.9
Olfactory groove	4	127.9 ± 98.8	36.2 ± 27.5	103.8 ± 73.0	23.7 ± 21.4
Suprasellar	2	18.0 ± 19.0	12.8 ± 9.8	10.1 ± 10.9	9.4 ± 9.9
Posterior fossa	10	69.2 ± 56.7	27.2 ± 25.4	67.9 ± 56.8	27.0 ± 26.9
Attachment to the sinus	1	114.1	68.8	111.9	34.9

Tumour volume in cm^3^

*TTV* total tumour volume; *TV* tumour volume

### Detection

**Fig. 2:**
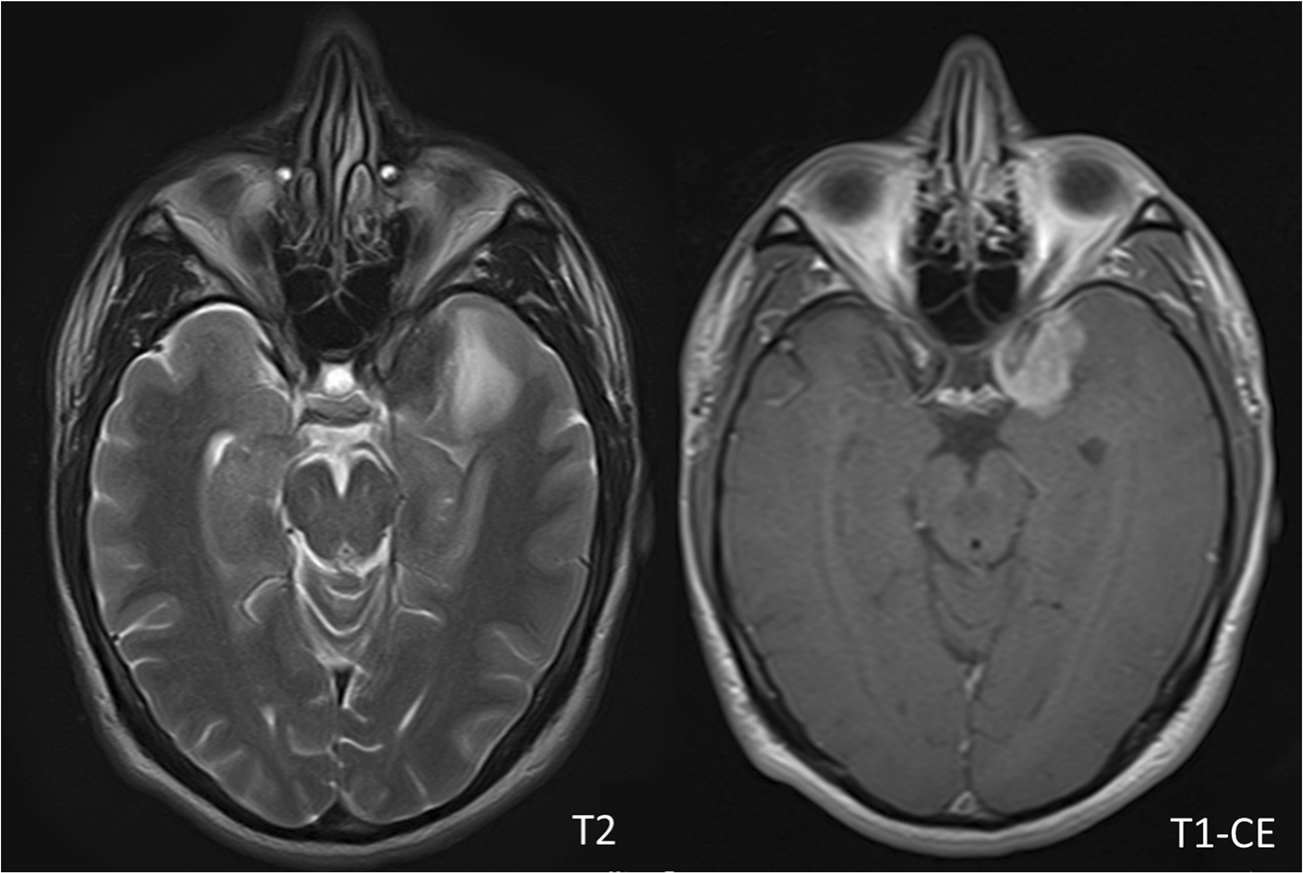
A 41-year-old man with a meningioma grade II located at the medial sphenoid wing. The meningioma showed a rather small tumour size and was visually rather easily detectable

In 55 of 56 patients the DLM detected presence of meningiomas, leading to a detection accuracy of 98%. The single meningioma that was not detected was a grade II meningioma located at the skull base (os sphenoidale) with a rather small tumour size of 12.7 cm^3^ and little surrounding oedema of 3.6 cm^3^. Visually, the tumour was well detectable (Fig. 2).

### Segmentation

Manual segmentation and automated deep-learning-based segmentation correlated well regarding TTV and contrast enhancing tumour volume. The mean Dice coefficient for TTV was 0.81 ± 0.10 (range, 0.46-0.93) and 0.78 ± 0.19 for T1CE tumour volume (range, 0.27-0.95). There was no significant difference between Dice coefficients of TTV and T1CE TV (*p* > 0.05). Further, Dice coefficients did not differ significantly between grade I and II meningiomas. For TTV, the mean Dice coefficient was 0.80 ± 0.11 for grade I and 0.83 ± 0.07 for grade II meningiomas. Mean Dice coefficient for T1CE tumour volume was 0.76 ± 0.21 for grade I and 0.83 ± 0.11 for grade II meningiomas.

**Fig. 3:**
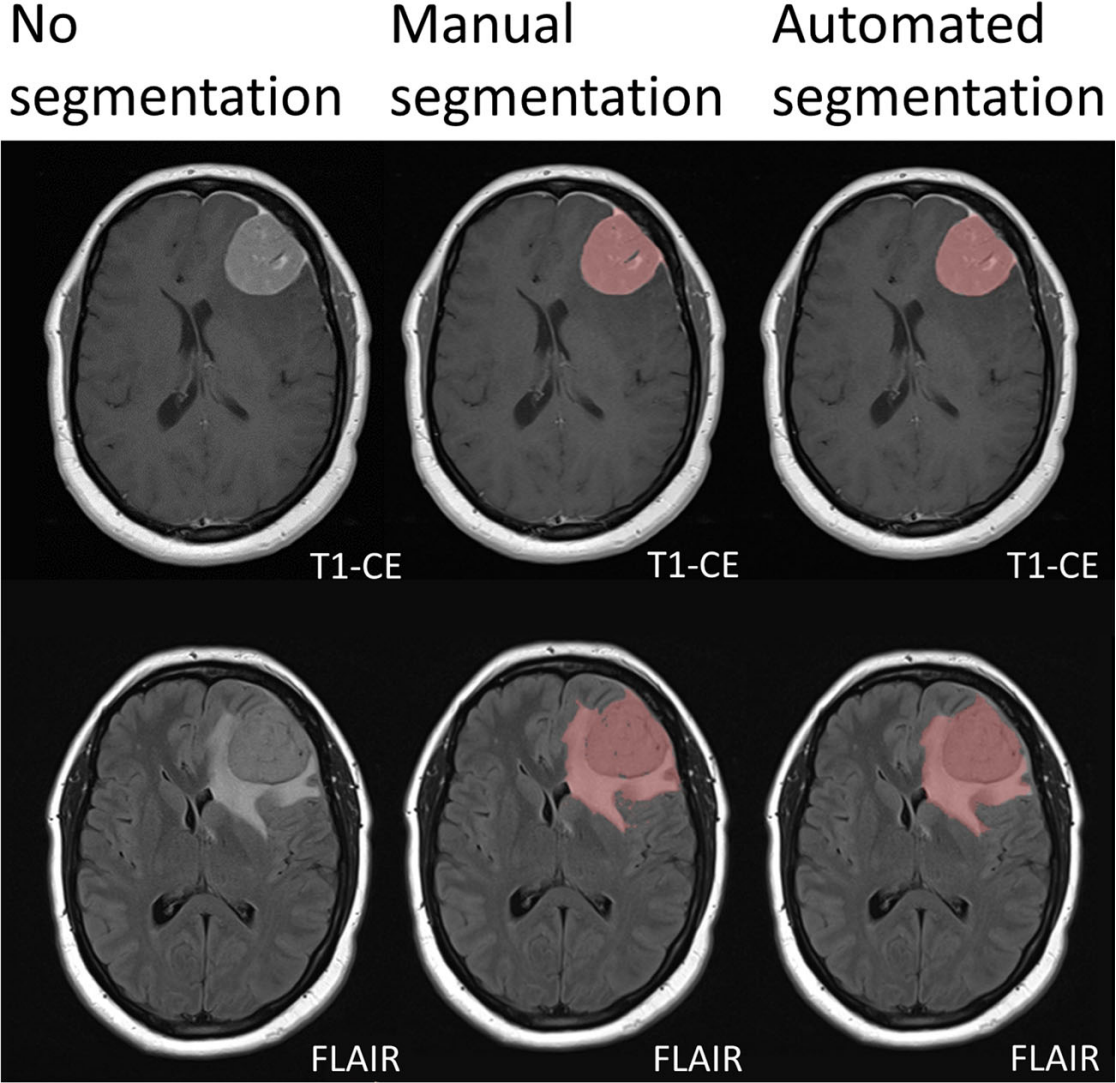
A 53-year-old woman with a meningioma grade II in the left frontal lobe with wide dural attachment. The meningioma is sharply circumscribed and shows strong gadolinium enhancement. Moderate to strong oedema of the surrounding white matter. The manual and automated segmentation correlate very well

In most cases DLM based automated segmentation worked well in one of the two defined tumour volumes, leading to high Dice coefficients (over 0.90) either for TTV or T1CE. In three patients Dice coefficients in both TTV and T1CE were 0.90 or better. A patient with a meningioma grade II in the left frontal lobe with surrounding oedema showed best automated segmentation with Dice coefficients of 0.92 for TTV and 0.95 for T1CE TV (Fig. 3).

**Fig. 4:**
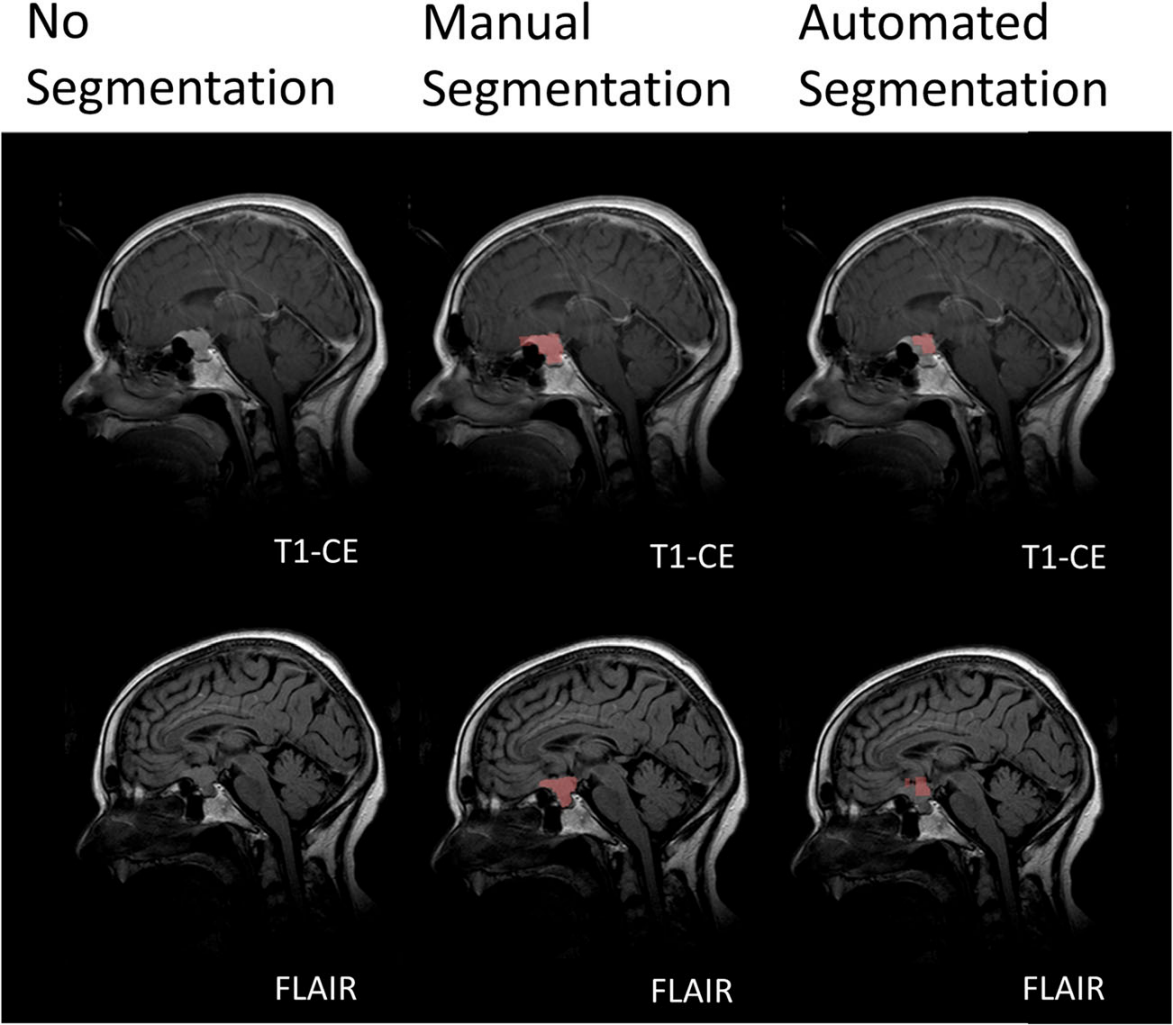
A 56 year-old woman with a meningioma I medial at the os sphenoidale next to the sella turcica. The meningioma showed a rather small tumour size without surrounding oedema

In some patients automated segmentation did not perform appropriately and resulted in Dice coefficients below 0.70. This was mostly the case either for TTV or T1CE tumour volume. Dice coefficients below 0.70 were obtained in three cases for TTV and nine cases for T1CE TV. Only in one patient with a small paramedian meningioma grade I at the os sphenoidale without surrounding oedema both TTV (0.52) and T1CE tumour volume (0.58) showed Dice coefficients below 0.70 (Fig. 4).

In the 16 meningiomas that were attached to the skull base, automated segmentation appeared to perform slightly worse than for meningiomas situated at the convexity of the skull, however without significance of differences. Mean Dice coefficients for TTV were 0.78 ± 0.12 for meningiomas at the skull base and 0.82 ± 0.08 for meningiomas situated at the convexity of the skull. For T1CE tumour volume mean Dice coefficients were 0.73 ± 0.23 at the skull base and 0.81 ± 0.15 at the convexity of the skull.

## Discussion

This study investigated automated detection and segmentation of meningiomas by DLM. The method proved to allow for accurate detection and segmentation, even though diverse MR images from different scanners, including data from referring institutions (66% of the available images) were included in this study. Detection accuracy was high (>98%). Automated segmentation correlated well with manual segmentations. Overlap measured by Dice coefficients was high for TTV and for T1CE TV (0.81 and 0.78, respectively). These results are comparable with other recently published studies in brain lesion segmentation using deep learning [14, 15, 28–31] and general segmentation accuracies accounting for intra- and inter-reader variabilities [14, 19]. Numerous deep convolutional neuronal networks with different technical specifications have been applied and tested for brain tumour segmentation [13, 26]. Gliomas are brain tumours with strong clinical implications and in focus of research for automated tumour segmentation. The results so far have been promising and even accurate differentiation between distinct tumour compartments were possible [13–15, 28, 29, 32–34]. Meningioma segmentation has also been a focus of research and several semi-automated and automated approaches have shown promising results in TV definition [23–25, 35]. One recent study investigated accuracies of TV definitions for longitudinal evaluation of meningiomas treated with stereotactic radiation. Segmentation accuracies for contrast enhancing tumour parts have been reported to be high, with Dice coefficients of 0.87; however, this approach used a manual pre-therapy segmentation for following post-therapy automated TV definition and therefore comparison of these results to our fully automated approach is difficult. Nevertheless, the study design from Shimol et al [25] has an excellent clinical focus and the workflow appears well suited for clinical meningioma surveillance as it contains accurate and consistent post-radiation measurements by the above-mentioned expert meningioma delineation in the pre-therapy scans and manual adaptions for clinical validation of the post-therapy segmentation data. It is well known that the extent of peritumoral oedema has a decisive impact on the clinical outcome as well as intraoperative performance. Volume definition therefore appears warranted and has been achieved by a semi-automated approach to a satisfactory level [23]. Semi-automated methods based on region growing and fuzzy clustering proved also to be feasible in unenhanced T1- and T2- weighted MR images [24].

**Fig. 5:**
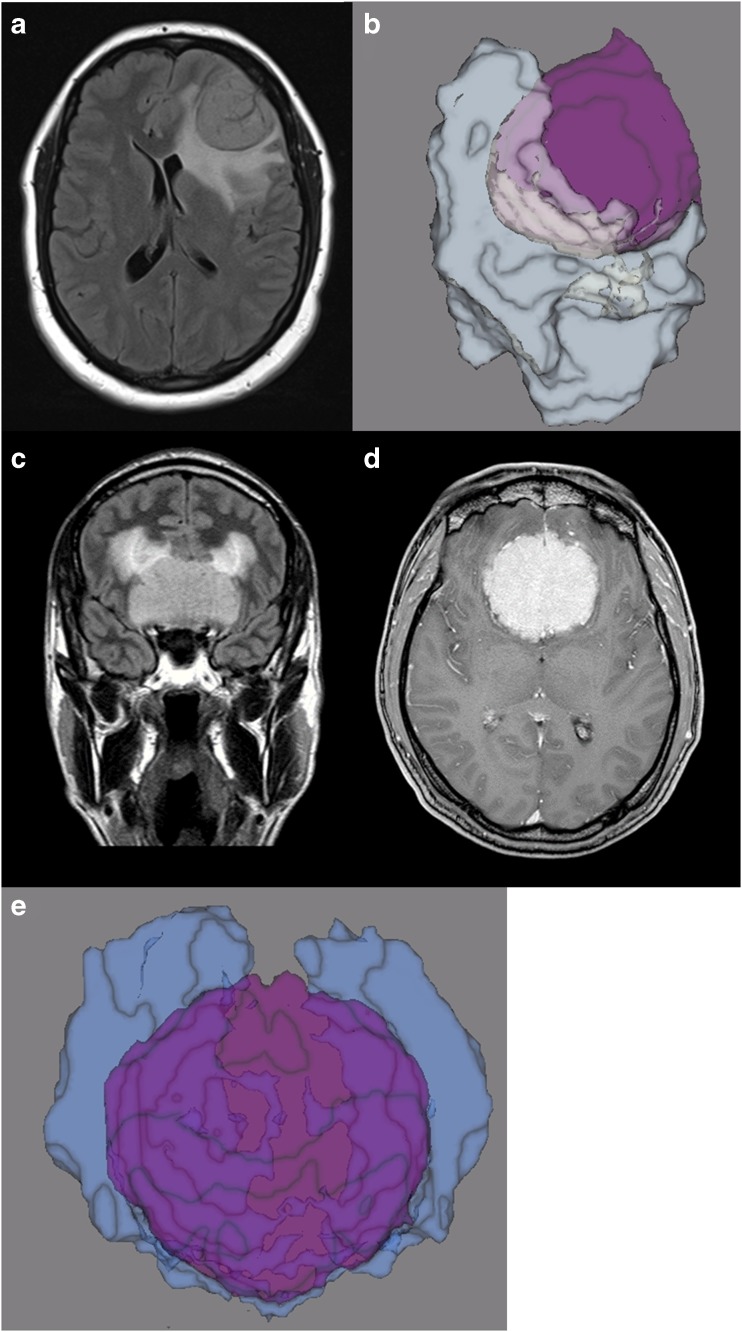
Three-dimensional rendering of two segmented tumour volumes. **a, b** Patient from Fig. 3, a 53-year-old woman with a meningioma grade II in the left frontal lobe with wide dural attachment. **c, d, e** A 33-year-old man with a grade I meningioma of the falx with great tumour volume in both frontal lobes and surrounding oedema in the adjacent white matter. Dice coefficients were 0.89 for TTV and 0.92 for T1CE TV. **a** and **c** FLAIR images; **d** T1CE MR image. **b** and **e** Three-dimensional rendering of the two meningiomas; contrast-enhancing tumour parts are displayed in *purple* and surrounding oedema in *white and blue*

The accurate automated detection of a cerebral tumour, as presented in our study, is clinically relevant as it allows for a preselection of lesions and patients of priority and as a control mechanism for the radiologist as a computer-assisted device [13, 20, 36–39]. Beyond this, the automated segmentation offers improved approaches to clinical assessment in the imaging routine, as it may allow for a more precise therapy planning (Fig. 5), enhanced detection and improved monitoring or additional evaluation of tumour features with radiomics approaches [1, 11–13, 40]. Further, automated detection allows for high reproducibility as it avoids inter- and intra-rater variability of tumour volume definition which has been reported as high [14, 19]. Reliable volumetric detection of tumour growth will allow for improved therapy decisions, as conventional diameter methods tend to underestimate tumour growth [1, 12]. Further, volumetric assessment in clinical routine is time-consuming, an automated segmentation is therefore warranted. The automated evaluation may further be transferred for the purpose of planning stereotactic radiation therapy and surgery [5, 13, 38, 41, 42].

The applied DLM was trained on glioma imaging data. Despite different appearances of the two tumour entities, detection and segmentation accuracies were high. Gliomas consist of contrast-enhancing tumour parts, necrosis and surrounding oedema and are quite varied. In contrast, meningiomas present as solid contrast-enhancing tumours with surrounding oedema and necrosis is less common. Meningiomas tend to show less complex tumour structures than, for example, glioblastomas and it could therefore be argued that classification accuracies should be higher. But there are several challenging aspects that make segmentation of meningiomas also difficult: (1) surrounding oedema in FLAIR may result in complex tumour structures, as can be seen in Fig. 5; (2) meningiomas are predominantly located in association with the dura and/or the skull base with presence of bordering hyperintense structures (e.g. dura, vessels) making delineation challenging; (3) FLAIR signal intensities differ strongly and meningiomas can even present isointense to normal brain tissue; (4) meningiomas and surrounding tissue also present heterogenous when oedema and necrosis are present next to contrast-enhancing tumour [5, 7, 20, 43, 44]. Therefore, the provided tumour detection and segmentation algorithm appears to be feasible for different cerebral tumour entities and even other brain lesions. Considering meningiomas of the skull base the applied DLM performed slightly worse than for convexity lesions. Even though this finding was not significant, it is important to consider as radiologists may need most assistance from a DLM for lesion segmentation in this area. Further, it needs to be discussed whether or to what extent intra-axial glioma training data might impact performance for the detection and segmentation of extra-axial meningiomas of the skull base. Thus, additional meningiomas as training data for the applied DLM may enhance future performance. The result should be a multifunctional detection and segmentation tool for neuro-oncology.

This study has several limitations that need to be considered beyond the retrospective study design. Since the study tests the segmentation of known meningiomas, the study does not test the accuracy for the detection of meningiomas in general. Also, it was not tested how far the DLM would segment false-positive tumour volume in normal brain MRI. Further, the presented segmentation accuracies between automated and manual segmentations might still be too preliminary for clinical applications and should be evaluated in future studies. The relatively small amount of cases may not reflect all types and sizes of meningiomas. The available image data were quite diverse as they included examinations from referring institutions. Further, gliomas were used as training data for the DLM; however, the study aimed to evaluate to what extent automated detection and segmentation was possible despite difference in training and despite heterogenic imaging data, as would appear in clinical routine. The study did not aim to present a DLM specifically designed for meningioma segmentation. Nevertheless, we plan to adapt the applied DLM with the manual meningioma segmentations as training data to yield improved results in future studies.

## Conclusions

Automated detection and segmentation of meningiomas based on a DLM were accurate and reliable. Precise and standardised TV definition allows for a more sensitive detection of tumour growth and thereby may improve monitoring and treatment of this highly frequent tumour entity. Further, automated detection by machine learning could function as a useful tool for pre-assessing and preselection as well as a control mechanism for radiologists.

## Electronic supplementary material

ESM 1 (DOCX 21 kb)

## Abbreviations

DLM: Deep-learning model
FLAIR: Fluid-attenuated inversion recovery
T1CE: T1-weighted contrast-enhanced MRI
TTV: Total tumour volume
TV: Tumour volume
