# Supplementary material for: Fully automated detection and segmentation of meningiomas using deep learning on routine multiparametric MRI
Source: Eur Radiol. 2018 Jun 25;29(1):124–32. doi: 10.1007/s00330-018-5595-8 (PMC6291436; doi:10.1007/s00330-018-5595-8)
Supplement: Supplementary file 1 — (DOCX 21 kb) [file 330_2018_5595_MOESM1_ESM.docx]

| **Supplementary Table 1 - MRI scan parameters** | | | | | | | | | | |
| --- | --- | --- | --- | --- | --- | --- | --- | --- | --- | --- |
|  |  | |  | |  | | |  | |  |
| **Philips Ingenia 3.0 Tesla** | | | | | | | | | | |
| **Parameter** | **FLAIR** | | **T1 SE** | | **T2 TSE** | | | **T1 SE CE** | | **3D T1 FFE CE** |
| Field of view (mm) | 250 x 189 | | 250 x 188 | | 240 x 199 | | | 250 x 188 | | 260 x 210 |
| Matrix | 280 x 189 | | 256 x 204 | | 428 x 271 | | | 256 x 204 | | 412 x 335 |
| Slice thickness (mm) | 5.0 | | 5.0 | | 5.0 | | | 5.0 | | 1.2 |
| Repetition time (msec) | 12000 | | 705 | | 4062 | | | 675 | | 9.6 |
| Echo time (msec) | 140 | | 13 | | 80 | | | 13 | | 4.8 |
| Inversion time (msec) | 2850 | |  | |  | | |  | |  |
| **Philips Ingenia 1.5 Tesla** | | | | | | | | | | |
| **Parameter** | **FLAIR** | | **T1 SE** | | **T2 TSE** | | | **T1 SE CE** | | **3D T1 FFE CE** |
| Field of view (mm) | 230 x 183 | | 230 x 183 | | 230 x 185 | | | 230 x 183 | | 220 x 186 |
| Matrix | 256 x 141 | | 256 x163 | | 384 x 246 | | | 256 x 163 | | 244 x 208 |
| Slice thickness (mm) | 5.0 | | 5.0 | | 5.0 | | | 5.0 | | 1.6 |
| Repetition time (msec) | 6000 | | 765 | | 7474 | | | 765 | | 25 |
| Echo time (msec) | 120 | | 15 | | 100 | | | 15 | | 7.8 |
| Inversion time (msec) | 2000 | |  | |  | | |  | |  |
| **Philips Achieva 3.0 Tesla** | | | | | | | | | | |
| **Parameter** | **FLAIR** | | **T1 SE** | | **T2 TSE** | | | **T1 SE CE** | | **3D T1 FFE CE** |
| Field of view (mm) | 250 x 189 | | 250 x 188 | | 250 x 209 | | | 250 x 188 | | 230 x 200 |
| Matrix | 280 x 160 | | 256 x 204 | | 436 x 283 | | | 256 x 204 | | 364 x 318 |
| Slice thickness (mm) | 4.0 | | 5.0 | | 5.0 | | | 5 | | 1.2 |
| Repetition time (msec) | 12000 | | 676 | | 3000 | | | 676 | | 8.5 |
| Echo time (msec) | 140 | | 13 | | 80 | | | 13 | | 3.9 |
| Inversion time (msec) | 2850 | |  | |  | | |  | |  |
| **Philips Intera 1.5 Tesla** | | | | | | | | | | |
| **Parameter** | **FLAIR** | | **T1 SE** | | **T2 TSE** | | | **T1 SE CE** | | **3D T1 FFE CE** |
| Field of view (mm) | 230 x 183 | | 250 x 177 | | 230 x 212 | | | 250 x 177 | | 200 x 179 |
| Matrix | 256 x 162 | | 280 x 138 | | 256 x 184 | | | 280 x 138 | | 224 x 199 |
| Slice thickness (mm) | 6.0 | | 6.0 | | 6.0 | | | 6.0 | | 1.6 |
| Repetition time (msec) | 6000 | | 582 | | 7808 | | | 582 | | 18 |
| Echo time (msec) | 100 | | 15 | | 110 | | | 15 | | 10 |
| Inversion time (msec) | 2000 | |  | |  | | |  | |  |
| **Range of scanner parameters of referring institutions (Siemens, Toshiba, Philips [1.0-3.0 Tesla])** | | | | | | | | | | |
| **Parameter** | **FLAIR** | | **T1** | | **T2** | | | **T1 CE** | |  |
| Field of view (mm) | n/a | | n/a | | n/a | | | n/a | |  |
| Matrix | 256-384 x 188-255 | | 256-512 x 179-310 | | 256-512 x 187-384 | | | 224-512 x 173-336 | |  |
| Slice thickness (mm) | 5-6 | | 5-6 | | 4-6 | | | 1.6-6 | |  |
| Repetition time (msec) | 6000-9000 | | 174-706 | | 2750-6757 | | | 18-787 | |  |
| Echo time (msec) | 97-140 | | 2.3-15 | | 80-121 | | | 2.4-18 | |  |
| Inversion time (msec) | | 2000-2500 | |  | |  |  | |  | |
| *FLAIR - Fluid attenuated inversion recovery; SE - Spin echo; TSE - Turbo spin echo; FFE - Fast field echo; n/a - Not available* | | | | | | | | | | |
